# Targeted Deep Sequencing of Mycosis Fungoides Reveals Intracellular Signaling Pathways Associated with Aggressiveness and Large Cell Transformation

**DOI:** 10.3390/cancers13215512

**Published:** 2021-11-02

**Authors:** Marion Wobser, Sabine Roth, Silke Appenzeller, Roland Houben, David Schrama, Matthias Goebeler, Eva Geissinger, Andreas Rosenwald, Katja Maurus

**Affiliations:** 1 Venereology and Allergology and Skin Cancer Center, Department of Dermatology, University Hospital Würzburg, 97080 Würzburg, Germany; wobser_m@ukw.de (M.W.); houben_r@ukw.de (R.H.); Schrama_d@ukw.de (D.S.); Goebeler_M1@ukw.de (M.G.); 2 Comprehensive Cancer Center Mainfranken, University Hospital of Würzburg, 97080 Würzburg, Germany; sabine_roth@uni-wuerzburg.de (S.R.); rosenwald@uni-wuerzburg.de (A.R.); 3 Institute of Pathology, University of Würzburg, 97080 Würzburg, Germany; silke.appenzeller@uni-wuerzburg.de (S.A.); geissinger@uni-wuerzburg.de (E.G.); 4 Pathology Practice, 85049 Ingolstadt, Germany

**Keywords:** mycosis fungoides, cutaneous T-cell-lymphoma, panel sequencing, large cell transformation, CD30

## Abstract

**Simple Summary:**

In order to decipher the molecular mechanisms of large cell transformation (LCT) in mycosis fungoides (MF), we screened 51 longitudinally obtained skin samples of mycosis fungoides patients (*n* = 27) with versus without large-cell transformation by the means of targeted deep sequencing in close clinicopathological correlation. The analysis of longitudinally obtained tissue revealed a dynamic mutational profile in the context of an evolutionary selection processes with the example of *PLCG1* alterations. In patients with an aggressive clinical course, we detected high mutational heterogeneity revealing the highest frequency of mutations in patients with LCT. The affected genes included members of the JAK/STAT signaling pathway and epigenetic modifiers. The key findings of our analysis included recurrent activating RAS mutations (*KRAS* and *NRAS*) being exclusively present in LCT MF; what is of note is that these molecular aberrations were already present in early stages; thus, *RAS* mutations in MF exhibit a prognostic marker for a higher risk of relapse and progression and restricted prognosis. As RAS mutated tumors are currently in the focus of novel targeted treatment options in several clinical trials, such personalized treatment modalities might offer novel therapeutic options for RAS mutated MF patients.

**Abstract:**

Introduction: Large-cell transformation (LCT) of mycosis fungoides (MF) has been associated with a higher risk of relapse and progression and, consequently, restricted prognosis. Its molecular pathogenesis has not been elucidated yet. Materials and Methods: In order to address molecular mechanisms of LCT, we performed hybrid capture panel-based sequencing of skin biopsies from 10 patients suffering from MF with LCT versus 17 patients without LCT including follow-up biopsies during clinical course, respectively (51 samples in total). The analyzed patients were attributed to three different groups based on the presence of LCT and clinical behavior. Results: While indolent MF cases without LCT did not show pathogenic driver mutations, a high rate of oncogenic alterations was detected in patients with LCT and aggressive clinical courses. Various genes of different oncogenic signaling pathways, including the MAPK and JAK-STAT signaling pathways, as well as epigenetic modifiers were affected. A high inter-individual and distinctive intra-individual mutation diversity was observed. Oncogenic RAS mutations were exclusively detected in patients with LCT. Conclusion: Our data demonstrate that LCT transition of MF is associated with increased frequency of somatic mutations in cancer-associated genes. In particular, the activation of RAS signaling—together with epigenetic dysregulation—may crucially contribute to the molecular pathogenesis of the LCT phenotype, thus conveying its adverse clinical behavior.

## 1. Introduction

Mycosis fungoides (MF), the most common lymphoma of the skin, is characterized by patches and plaques that may evolve into cutaneous tumors during further disease course in case of progression. While the prognosis of MF is excellent in early skin-limited stages, the occurrence of tumors and/or systemic dissemination relative to nodal or visceral sites confers a restricted disease-specific survival [1,2]. The classical histological hallmarks of MF are epidermotropic small-sized to medium-sized atypical lymphocytes with condensed chromatin structures. Neoplastic lymphocytes of larger cell size exhibiting a vesicular nucleus—thus harboring cytological features of so-called large cell transformation (LCT)—may be intermingled within the infiltrate [3]. Importantly, it has been demonstrated that cells of “normal” MF and large cell lymphoma of a given patient share the same clonal origin [4,5]. According to previous studies, decisive cut-off values for fulfilling the sensu strictu criteria of LCT of MF have been established and consented as the presence of more than 25% neoplastic cells of larger cell size (i.e., four times the diameter of a normal small lymphocyte) within the infiltrate [3,6,7,8]. LCT can be observed with variable frequency in about 10–20% of cases across all stages of MF; however, it is more common (about 50%) in advanced disease such as in patients with skin tumors or nodal dissemination [9,10]. The cumulative probability of transformation over a time span of 4 years and 12 years has been estimated to reach up to 21% and 39%, respectively [10].

The presence of LCT of MF has been associated with a higher risk of relapse and progression and consecutively a restricted disease-specific and/or overall survival [2,3,10,11,12,13,14,15,16] especially when occurring in extra-cutaneous sites such as lymph nodes. Although the phenomenon of LCT in MF over decades has been within the scope of a plethora of descriptive studies mainly focusing on clinicopathological correlations, its molecular pathogenesis still remains widely elusive up to now. What is of note is that the occurrence of LCT in MF is often associated with increased surface expression of CD30, a receptor protein of the tumor necrosis factor receptor (TNFR) family. This raises the question as to whether there might be a common underlying molecular mechanism linking both events. 

In order to address this question and to elucidate potential underlying molecular mechanisms driving LCT and/or CD30-upregulation of neoplastic cells of MF, the mutational pattern of MF with versus without LCT was assessed by sequencing a panel of genes that have been described to be affected in MF and further lymphomas. Obtaining repetitive biopsies during the patients’ clinical course over a long follow-up period enabled us to approach this issue both in an inter-individual as well as an intra-individual comparative setting. In addition, sequential biopsies enabled us to scrutinize any predictive issues of the detected genetic variants. 

## 2. Materials and Methods

### 2.1. Patient Selection and Characteristics

We sequenced 51 tissue samples (skin *n* = 50, lymph node *n* = 1) from 27 patients with MF including blood samples of all patients as matched controls. The analyzed patients were attributed to three different groups based on the presence of LCT and clinical behavior. Group I included the samples of 10 patients (patients #1–10) suffering from CD4-positive MF who—either at time of first diagnosis or during further course of disease (follow-up time 73 months and range 36–156 months)—exhibited LCT in at least one cutaneous (patch/plaque/tumor) or non-cutaneous (nodal) lymphoma manifestation. Within group I, all patients presented or progressed to higher stage ≥ IIB with the exception of patient #10. Sequential tissue samples obtained over the disease course were available and analyzed in 6 out of these 10 patients exhibiting variable CD30-expression levels. In 4 of these, 6 patients samples with versus without LCT were available, which were obtained either at the same time point from different lesions or as sequential biopsies during the clinical course. Moreover, we analyzed, as a comparator, additional samples of patients who did not exhibit LCT at any time point (*n* = 17) during their longstanding clinical course (follow-up time 132 months and range 22–348 months); repetitive biopsies were again available in 7 out of these 17 patients. Within this latter group, both patients with an “aggressive course” (*n* = 10; patient #11–20), i.e., who later progressed to higher stages ≥ IIB (group II), and patients with an “indolent” disease course (long-standing MF confined to stage IA/B over years) (*n* = 7; patient #21–27) (group III) were compiled. Patients with other cutaneous T-cell lymphoma (CTCL) subtypes such as Sézary syndrome (SS) or CD8-positive MF variants were not included in order to keep the cohort clear. 

All patients were diagnosed at the Department of Dermatology, University Hospital Würzburg, and the Institute of Pathology, University of Würzburg. Approval of the entire study was obtained from the Ethics Committee at the Medical Faculty of the University of Würzburg, Germany (vote number 115/15). Informed consent was obtained prior to investigations. Staging examinations at primary diagnosis and during follow-up as well as lymphoma classification followed the criteria proposed by the International Society for Cutaneous Lymphomas and the European Organization of Research and Treatment of Cancer [17,18]. Treatment was performed according to national and international guidelines [19] including the application of anti-CD30 antibody brentuximab vedotin for CD30-positive cases since its approval in Germany in the year 2018. Other treatment modalities comprised—adjusted to tumor stage, disease dynamics and comorbidities—topical steroids, UV light therapy, radiation and different systemic treatments such as methotrexate, bexarotene, interferon and gemcitabine. 

Only patients with a close follow-up of at least 20 months (mean 111 months, range 22–348 months) were considered for further investigation. Follow-up biopsies of successive lymphoma manifestations during disease course with repetitive histological examination and investigation were available for 13 of the included patients. Detailed patient characteristics including data on disease evolvement and survival in correlation to molecular data are enlisted in Table 1. Data on treatment modalities are summarized in Supplementary Table S1. In addition to the enlisted therapies, all patient received topical steroids.

### 2.2. Clinicopathological Assessment

Histological diagnosis of respective biopsies was confirmed by 3 (dermato-) pathologists of the Department of Dermatology, University Hospital Würzburg (MW), and the Institute of Pathology, University of Würzburg (EG, AR). Only samples with at least 5% tumor cell content were investigated. The presence of LCT was determined as previously defined [3,6,14,18]. In short, LCT was taken for granted if >25% of the infiltrate consisted of large or overt blastic tumor cells either in a diffuse pattern or forming microscopic clusters. The cytological category of a large cell was provided as fulfilled if the cell size was 4 times larger than of a normal small lymphocyte. If CD30 expression of lymphoma cells was higher than 10%, the sample was designated as CD30-positive. 

Immunohistological analysis included—among others—staining for T-cell antigens (CD2, CD3, CD4, CD5, CD7 and CD8), B-cell antigens (CD20 and CD79a) and histiocytes (CD68) as well as assessment of CD30-expression (CD30). Any coexistence of lymphomatoid papulosis (LyP) (presence of spontaneously regressing papules) or cutaneous anaplastic large cell lymphoma (cALCL) (occurrence of large non-healing tumors without preexisting patches or plaques) as based on close clinicopathological correlation was excluded in order to prevent putative blurring of data; such cases have been published elsewhere by our group [20,21].

Survival data were analyzed by using the Kaplan–Meier method. Survival curves were compared using the log-rank test. Overall survival was calculated from time of first diagnosis of MF. All causes of death were included into the survival analysis. Comparison of different groups with respect to the detected mutations was performed by the Fisher´s exact test (Supplementary Table S2). Statistical analysis was performed using GraphPadPrism statistical software (Version 3.02, San Diego, CA, USA). 

### 2.3. Sample Processing

Genomic DNA extraction, library preparation and sequencing were performed as previously described [20]. 

#### 2.3.1. DNA Extraction

Genomic DNA from tissue specimen and the corresponding blood samples were extracted with the DNeasy Blood and Tissue Kit (Qiagen, Hilden, Germany). DNA quantitation was assessed by Qubit dsDNA Broad Range Assay (Life Technologies, Darmstadt, Germany).

#### 2.3.2. Hybridization Based Panel Sequencing

HaloPlexHS Target Enrichment System (Agilent Technologies Inc., Santa Clara, California, USA) was used for library preparation strictly according to the manufacturer’s protocol. The captured libraries were amplified during 23 PCR cycles. The libraries were sequenced on the MiSeq platform with a 150 bp paired-end sequencing approach (Illumina, San Diego, CA, USA).

### 2.4. Bioinformatical Data Analysis

Quality trimming, read alignment and somatic variant calling were performed as already published [21] and presented in Supplementary Table S3. All variants were visually examined by using the Integrative Genomics Viewer v2.3.68 [22]. Additionally, all detected variants were verified in all corresponding samples of the same patient. All detected variants are shown in Supplementary Table S4. The variant allele frequency was set to 2%. Somatic variants were reported if the variant resulted in a protein alteration or affected a splice site (single nucleotide variants and indels that were not present in the matched control sample).

## 3. Results

In order to address the molecular mechanisms of LCT, we performed hybrid capture panel-based sequencing of tissue biopsies obtained from 10 patients with LCT and compared them with those from 17 patients without LCT including follow-up biopsies during the clinical course, respectively. Overall, 51 samples were sequenced in total. The applied panel includes full-length coding regions of 40 lymphoma-associated genes [20]. As LCT implies a more aggressive clinical course within the entity of MF, we designed our study with three independent patient groups. By performing this, we were able to compare aggressive MF—either with or without LCT—versus non-aggressive/indolent MF. The groups were named as following: group I—aggressive LCT-MF; group II—aggressive MF without LCT; group III—indolent MF).

### 3.1. Significantly More Patients with Aggressive MF Show Mutations in Lymphoma-Associated Genes 

**Figure 1:**
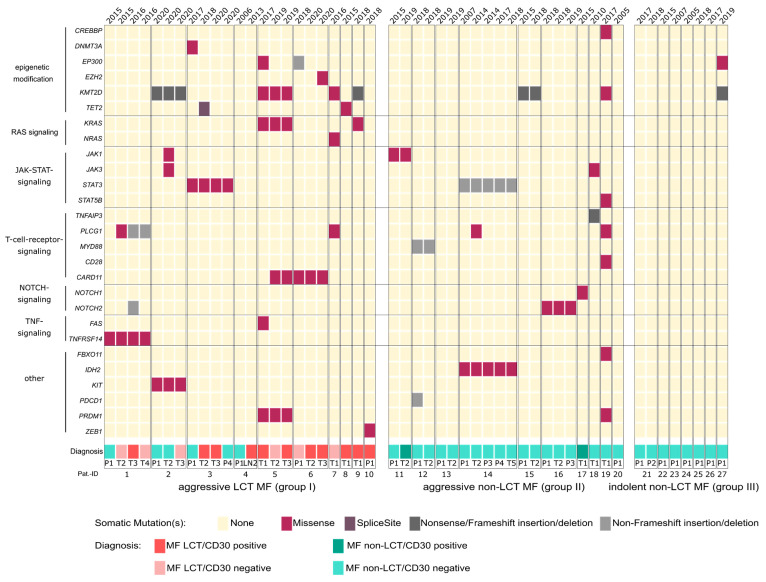
Mutational profile of LCT MF. Sequenced patients are divided into three groups: group I, aggressive LCT-MF (large cell-transformed mycosis fungoides); group II, aggressive non-LCT MF; and group III, indolent non-LCT MF. Groups II and III served as control cohorts. Different intra-individual samples are illustrated chronologically. Sequenced genes and their functions are annotated at the left. Type of mutations and diagnoses are color-coded as indicated. T: tumor stage of MF; P: patch/plaque stage of MF; LN: lymph node.

Our analysis revealed alterations in 27 of the investigated genes, whereas 13 genes were unaffected. We detected 79 protein-altering mutations in total. Within the cohort of indolent small-cell MF (group III), only one of the seven analyzed patients (14%) showed any mutation (patient 27: two mutations in total). Out of the remaining 20 patients with an aggressive clinical course (groups I and II), significantly more patients harbored at least one mutation of any of the analyzed genes (*p* = 0.0017). Within the group of aggressive MF, 17 patients (85%) harbored mutations in the genes of our panel. Importantly, the highest frequency of mutations was observed in MF patients with LCT (group I) (Figure 1). With respect to biomolecular effects of the detected mutations, we assume non-functional proteins as a consequence of the identified nonsense and frameshift mutations. The molecular function of several of the additionally detected missense mutations is, however, currently unknown. All relevant mutations with respective biological implications are explained in the subsequent paragraphs of the manuscript.

### 3.2. Mutational Heterogeneity in Patients with an Aggressive Clinical Course

Patients with aggressive MF show a heterogeneous mutational profile, which points to a distinctive inter-individual diversity of this lymphoma subtype. Various genes of different oncogenic signaling pathways, including the MAPK and JAK-STAT signaling pathways, as well as epigenetic modifying regulators were affected (*DNMT3A, TET2, EZH2, EP300, CREBBP* and *KMT2D*). Such alterations displayed varying combinations thereof. Moreover, a distinctive intra-individual mutation diversity was observed in several patients. On the one hand, different samples obtained from the same patient shared identical unifying alterations (a) irrespective of lesion morphology (patch/plaque/tumor) and (b) independent of the respective time point of biopsy. On the other hand, however, both novel mutations not observed in earlier biopsies as well as—vice versa—lack of former mutations detected in the initially excised tumors were observed in several patients. These findings could in part be correlated with morphological and/or immunophenotypical changes, i.e., the advent of LCT and/or the acquisition of CD30 expression, as well as clinical features with respect to disease progression (Figure 1). 

### 3.3. Acquisition of PLCG1 Activating Mutations Is Associated with LCT Transformation in One Patient

**Figure 2:**
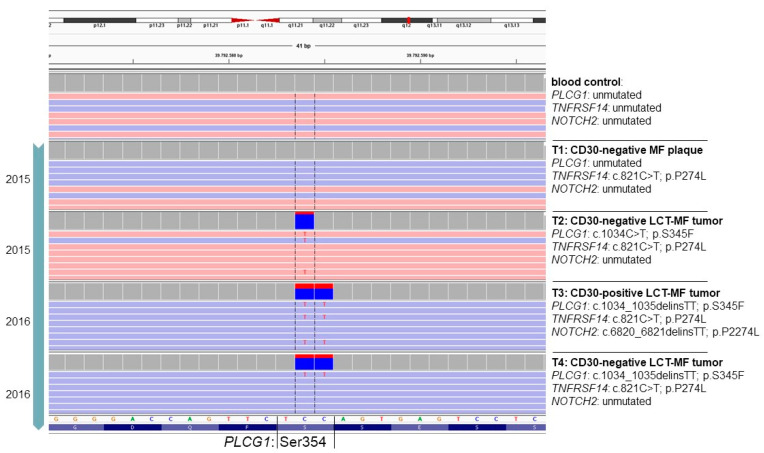
Intra-individual and intra-genic genetic heterogeneity of patient #1 during clinical course. Illustration of the genetic profile of the *PLCG1* hotspot Serine 354 in IGV (Integrative Genomic Viewer) of four different MF samples over time and the patient´s unmutated control tissue (blood). Moreover, additionally occurring mutations in *TNFRSF14* and *NOTCH2* are indicated on the right side along with LCT and CD30 status.

As a representative example, data on the mutational profile of patient #1 are illustrated in Figure 2. The occurring mutations constitute C > T or CC > TT transitions indicating a typical UV signature [23,24]. The depicted findings elucidate the complex genomic pattern of repetitive biopsies obtained from patient #1. The mutational pattern implies a link of the detected mutations to lesion type (plaque versus tumor) and the presence of LCT as well as CD30-expression. In patient #1, three different genes are mutated in any of the four analyzed samples, i.e., *PLCG1*, *TNFRSF14* and *NOTCH2,* albeit in different combinations. All samples share identical *TNFRSF14* missense mutation irrespective of lesion type (plaque/tumor) being conserved over a time course of 12 months. However, only in tumoral lesions with LCT is a *PLCG1* hotspot mutation present. Intriguingly, at the nucleotide level, the *PLCG1* hotspot Ser345 is altered in the three affected tumor samples in two different ways (c.1034C>T vs. c.1034_1035delinsTT), both resulting in the same amino acid exchange (p.Ser345Phe) with known oncogenic properties [25]. Recurrent associations of a specific differentiation step with a specific protein alteration that, however, might be caused by two independent genetic events suggest a causal relationship, as two different mutations were detected (c.1034C>T (point mutation) vs. c.1034_1035delinsTT (indel: deletion of two cytosines and insertion of two thymidines at position 1034 and 1035 in the coding sequence of the *PLCG1* gene). Therefore, it appears likely that the PLCG1^S345F^ mutation might contribute to LCT transformation in the MF of this patient. Of note, an additional *NOTCH2* mutation was only present in a tumor biopsy with CD30-expression of large-cell transformed lymphoma cells; this mutation was otherwise absent in all further CD30-negative samples. These observations strongly point to evolutionary selection processes.

### 3.4. JAK/STAT Mutations as Potential Surrogate Marker for Increased Tumor Aggressiveness 

A more detailed examination of oncogenic signaling pathways revealed an enrichment of genetic alterations within the JAK-STAT signaling pathway in MF patients with aggressive clinical course. Corresponding mutations were detected in *JAK1* and *JAK3* as well as the *STAT3* and *STAT5B* genes. Six of the twenty patients of groups I and II were affected. *JAK/STAT* mutations were restricted to groups I and II and were not present in the samples obtained from patients with an indolent clinical course of MF (group III). With one exception (*STAT5B*, p.G452A), all of the herein detected *JAK/STAT* mutations constitute hotspot mutations, which result in an already proven gain-of-function (*STAT3* p.Tyr640Phe and p.Glu616del; *JAK3* p.Met511Ile and p.Ala573Val; and *JAK1* p.Gly1097Val and p.Ser646Phe) [26,27] (Supplementary Table S2). Therefore, in this regard, JAK/STAT mutations might constitute potential surrogate markers for a more aggressive clinical course. In our cohort, however, the presence of JAK/STAT alterations was not indicative of LCT or CD30 expression in the individual biopsy samples, and due to limited patient number, this finding did not reach statistical significance for the above-mentioned parameters (*p* = 0.15)

### 3.5. Recurrent Activating RAS Mutations Are Exclusively Present in LCT MF Patients

By searching for relevant genetic aberrations that discriminate between patients with and without LCT, we identified one oncogenic signaling pathway that is exclusively altered in patients with LCT (*p* = 0.041). In three of the ten patients with LCT (patients #5, #7 and #9), we observed gain-of-function mutations in genes coding for RAS family members. Two of the three respective patients had the same pathogenic hotspot mutation in exon 2 of the *KRAS* gene (patients #5 and #9: p.Gly13Cys), whereas the third patient harbored a pathogenic hotspot mutation in exon 2 of *NRAS* (patient #7: p.Gly13Asp) (Supplementary Table S2). 

Similar oncogenic alterations in *KRAS*, *NRAS* and *HRAS* genes are strong causative drivers in a broad variety of solid and hematological tumors with mostly aggressive behavior [28]. Owing to the fact that *RAS* driver mutations were exclusively and recurrently detected in patients with LCT, our data suggest that activation of RAS signaling may play a crucial role in the process of LCT. However, *RAS* mutations were not related to the CD30 expression status (Figure 1).

In order to elucidate the link between *RAS* mutations and LCT in more detail, we scrutinized further sequential samples from patients #5 (*n* = 8) and #9 (*n* = 6) by Sanger sequencing/amplicon-based panel sequencing for the respective hotspot mutations within the *KRAS* gene. These samples included sequential biopsies obtained from patches, plaques and tumors during disease evolution with or without LCT/CD30-expression. Over a time course of seven and eight years, respectively, both patients exhibited the same *KRAS* alterations in all analyzed samples irrespective of biopsied lesion type, immunophenotype, cytological features or time point during disease. 

### 3.6. Genetic Alterations in Epigenetic Modifier Genes Are Overrepresented in MF with LCT

Mutations affecting genes coding for epigenetic modifiers were recurrently detected in nearly half of the analyzed patients of groups I and II. While in 9 out of 20 patients (45%) with aggressive MF harbored such mutations, only one patient with indolent MF was found to be mutated in the *KMT2D* gene (*p* = 0.204) (Figure 1). Moreover, the observed mutations within genes encoding epigenetic modifiers were clustering in patients with LCT; 7 out of the 10 patients (70%) of group I exhibited mutations, including deleterious alterations (nonsense and frameshift mutations) in at least one of the assessed genes encoding for the DNA and histone modifiers *DNMT3A*, *TET2*, *EZH2*, *EP300, CREBBP* and *KMT2D*. In total, 14 of the 20 different detected mutations within epigenetic modifiers were found in group I with significantly higher frequencies in comparison to non-LCT cases (*p* = 0.013). Within individual patients with repetitive biopsies, mutations affecting *KMT2D* were stably observed in all samples over time and were invariably present irrespective of clinical (patch/plaque/tumor) and histological features (cytology and CD30 expression). In contrast, the mutation statuses of the other epigenetic modifiers were highly variable between different biopsies obtained from individual patients. Interestingly, all *RAS*-mutated cases also exhibited a *KMT2D* mutation. 

### 3.7. Adverse Clinical Course in MF with LCT

**Figure 3:**
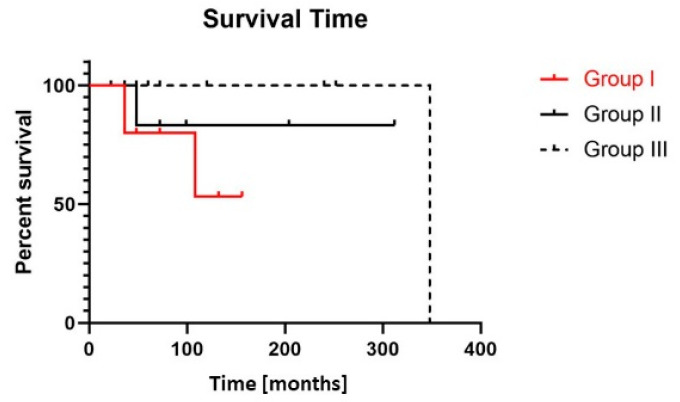
Patients with LCT show a reduced overall survival. Overall survival (Kaplan–Meier curve) of patients of groups I (MF with LCT) (*n* = 10), II (aggressive MF without LCT) (*n* = 10) and III (indolent MF without LCT) (*n* = 7) from date of first diagnosis of MF. Overall survival of group I versus II *p* = 0.4 and group I versus III *p* = 0.06 (log-rank test).

Although the initial stage at the time point of first presentation was comparable between groups I and II and both groups comprised patients with aggressive clinical behavior (progression to ≥stage IIB), only patients with LCT incurred death of lymphoma (*n* = 3) during the observation period and, altogether, presented the worst overall survival (Figure 3). For patients undergoing LCT at any time point during disease evolution (group I), mean survival from the timepoint of first diagnosis of MF was 73 months (range 36–156 months) versus mean survival for group II of 123 months (range 36–312 months) and for group III 145 months (range 22–348 months) (group I versus group III: *p* = 0.06). All patients with aggressive clinical course were similarly treated according to national/international guidelines by taking into account clinical presentation, stage, comorbidities and previous treatment approaches. 

In summary, our data demonstrate that the LCT transition of MF is associated with increased frequency of somatic mutations in tumor-promoting genes. In particular, activation of RAS signaling—together with epigenetic dysregulation—may crucially contribute to the molecular pathogenesis of the LCT phenotype conveying adverse clinical behavior.

## 4. Discussion

Although the negative prognostic implication of LCT in patients with MF and SS has been outlined in various independent clinical investigations, the underlying processes driving the eponymous cytological changes and the adverse biological behavior of large-cell transformed CTCL have not been elucidated up to this point in time. 

Recently, transformation of chronic lymphocytic leukemia to diffuse large B-cell lymphoma (Richter´s transformation) could be attributed in a comprehensive experimental setting relative to the activation of Akt signaling via Notch1 [29]. Similar extensive and functional approaches would be desirable with respect to LCT in CTCL in order to understand its underlying molecular mechanism, to better predict disease progression and, thus, apply risk-adapted and personalized treatment strategies in the future.

Previous investigations addressing large-cell transformed CTCL merely focused on single markers and their clinicopathological correlation and provided a rather heterogeneous portfolio thereof [7,11,30,31,32,33]. A close interaction with an inflammatory or tolerogenic tumor micromilieu was attributed to additionally contribute to the transition from an indolent to an aggressive state [34,35]. Recent deep sequencing approaches did not reveal a conclusive mutational pattern underlying LCT putatively owing to variable methodological procedures (whole exome sequencing versus targeted sequencing), heterogenous sampling (blood versus tissue) and—last but not least—different analyzed CTCL subtypes (MF versus SS versus CD30-positive lymphoproliferations) [36,37,38,39]. Fundamental differences between MF, SS and CD30-positive lymphoproliferations with respect to the cell of origin, the immunophenotype and the genomic profile have, however, been delineated over the last years [20,40,41]. In our study, we therefore focused on tissue samples (51 skin biopsies) obtained from classical clear-cut CD4-positive MF with well-characterized clinical course and histology and excluded any MF variants and any ambiguous borderline cases of CD30+ lymphoproliferations as well as SS. 

According to previously reported data [36], a higher overall mutation rate is observed in samples with LCT obtained from blood or skin in comparison to non-transformed CTCL. In line with these findings, we also detected the highest frequency of molecular alterations in samples of MF exhibiting LCT. 

As the pivotal finding of our targeted sequencing approach, we were able to detect recurrent activating *RAS* mutations (*NRAS* and *KRAS*) exclusively in patients with LCT (aggressive course) and not in patients without LCT (aggressive or indolent course). While the constitutive activation of the MAPK signaling pathway—as a consequence of active RAS—is an important oncogenic driver in solid tumors, it is rather uncommon in most hematological neoplasms [42,43]. With respect to CTCL, mutations in *RAS* and *MAPK* genes have only been rarely reported (<5% of analyzed cases). Somatic mutations in *BRAF* (p.Asp594Asn) [44], *KRAS* (p.Gly13Asp) [37] and *NRAS* (p.Gly13Cys and p.Gln61Lys) [37,39] have each been identified in one single case of SS, respectively. Concerning MF, activating mutations in *NRAS* (p.Lys117Glu) [36] and *KRAS* (p.Gly13Asp) were present once each [36,39]. With respect to our interests and for corroborating our findings, two of the above mentioned RAS-mutated SS cases as well as the previously reported *NRAS*-mutated MF case were designated as being large-cell transformed on histology—albeit not being stressed by the authors of the published reports [36,37]. Although information on LCT status was not provided for the other cases with oncogenic aberrations in MAPK pathway genes in the publication by Kiessling et al., the respective patients of this publication all presented at higher stages and displayed reduced overall survival [39]. One explanation might be that the observed genetic alterations of *RAS* genes only come along with a more advanced disease due to the inherent capacity of RAS downstream signaling to drive more aggressive biological behaviors. With this respect, all patients of our cohort with oncogenic alterations within the MAPK pathway (*KRAS* and *NRAS*) presented skin tumors at any time point during disease course, and the *NRAS*-mutated patient progressed from extensive tumor stage to systemic dissemination. However, readily available sequential biopsies of the *KRAS*-mutated patients showed respective genetic alterations even at an early stage of disease and in non-transformed lesions (patches/plaques) (irrespective of time point of biopsy) (data not shown), hence, harboring strong predictive issues. Therefore, our data imply that respective mutations in MAPK genes obviously impact the (further) development of LCT in that no other patient with aggressive MF but without LCT (group II) exhibited any analogous aberrations. 

Owing to the fact that LCT is associated with high risk progression and failure with respect to conventional therapy, targeting such *RAS* alterations emerges as a feasible innovative therapeutic approach to tackle the unmet clinical challenge. However, so far, no approved targeted therapy is available for the herein detected *RAS* mutations. Targeting *KRAS* p.Gly12Cys-mutations has recently been evaluated in non-small cell lung cancer (NSCLC) achieving durable clinical responses [45]. Disabling activated *KRAS* signaling in solid tumors by means of inhibiting cross-linking pathways/interfering molecules such as SHP2, MEK1/2, geranylgeranyl transferase-1 or ERK1/2 is currently being investigated in various clinical trials. Such approaches might also offer novel therapeutic options for *RAS*-mutated MF patients. 

The second pivotal finding in the LCT group included recurrent alterations in genes encoding for epigenetic modulators and modifiers (*TET2, DNMT3A, KMT2D, EP300, EZH2* and *CREBBP*) strongly clustering with group I. As epigenetic regulators control chromosomal structure and thereby transcriptional activity [46], it is conceivable that such aberrations might contribute both to the morphological changes as well as altered biological behavior observed in large-cell transformed MF. In fact, mutations in epigenetic regulators have been associated with an altered and open chromatin structure in several systemic B-cell and T-cell neoplasias [47]. The observation that *KMT2D* mutations were conserved over a long time span—as evidenced by the analysis of repetitive biopsies of patients with LCT—underscores its pivotal function for tumor biology. In addition, the known association between deranged epigenetic regulation and genomic instability might explain the highest mutational load in group I and the worst clinical behavior. Of note, all of the above described cases with *RAS* alterations exhibited additional *KMT2D* mutations, suggesting a mutually enforcing or synergistic mechanism. In this light, the transforming capacity of oncogenic RAS has been recently deduced to a close interplay with epigenetic gene silencing and remodeling of chromatin structure [48,49,50]. This opens up the field for the clinical application of inhibitors of kinases and epigenetic modifiers. With this respect, histone deacetylase inhibitors (HDACi) have been proven effective in CTCL and are FDA-approved for the treatment of MF and SS [51,52].

Some of these molecular alterations in chromatin modifiers such as TET2 were herein described for the first time in MF. Genome-wide sequencing and methylation analysis has shown rare mutations in *TET1*, *TET2* and *TET3* and genes further coding for chromatin-modifying proteins [44,53] (e.g., *DNMT3A, KMT2D, KMT2C* and *ARID1A*) in SS proceeding along with aberrant DNA methylation patterns [54]. In various non-cutaneous hematological tumors of different lineages, somatic mutations in *TET* or *IDH* genes or respective protein downregulation have been related to the loss of 5-hmC expression [53,55]. While similar investigations in SS are still lacking, in MF (including cases with LCT), reduced levels of 5-hmC were previously—as likewise observed by us—not associated with mutations or altered gene expression of *TET2* or *IDH* [33,38]. 

The longitudinal exploration of repetitive biopsies obtained from the same patients during their clinical course over a remarkably long follow-up adds to the peculiar strength of our study. By taking advantage of such an approach, we were able to identify the same genetic alterations present already in “pre-LCT” samples obtained at earlier time points before the occurrence of LCT within the same patient. Therefore, our data convey predictive issues of the identified genetic alterations with respect to LCT.

Although little is known about the regulatory mechanism of CD30 surface expression in CTCL, constitutive signaling via JAK/STAT might indeed confer one possible method to upregulate CD30 expression in MF due to STAT-responsive elements within the CD30 promotor [56]. In CD30-positive cutaneous lymphoproliferations (LyP and cALCL), we recently identified highly recurrent genetic alterations within the JAK/STAT pathway (SNVs and fusions) as the molecular driving force of CD30-positive LPD [20]. However, our data imply that additional genetic or epigenetic mechanisms beyond *JAK/STAT* mutations—by the means of further mutations, gene fusions or exogenous stimuli—appear to be necessary for finally orchestrating cell morphology and CD30 expression in MF and for shaping the final phenotype [21]. In our patient cohort, the presence of oncogenic *JAK/STAT* mutations was associated with a more aggressive clinical course. Currently, there is no approved biomarker stratified therapy with JAK inhibitors available. Nevertheless, several JAK inhibitors are approved or investigated in clinical trials concerning different diseases, such as ruxolitinib for the treatment of myelofibrosis and hydroxyurea resistant or intolerant polycythemia vera or itacitinib for treatment of acute graft-versus-host disease [57,58]. Preclinical models imply beneficial effects in JAK mutated tumors including cutaneous T cell lymphoma and might, therefore, pave the way for innovative treatment options in the future [59].

## 5. Conclusions

To summarize, we were able to pinpoint convergent molecular alterations within RAS genes and genes encoding chromatin modifiers as being associated with and/or predictive of LCT in intrinsically genetic heterogeneous MF. The predictive value of our data set may convey immediate impact for the clinical management in that MF patients with the above described genetic alterations might profit from closer disease monitoring or more aggressive (or in the future targeted) therapies due to their putative higher risk for the development of prognostically adverse LCT during further disease course. Therefore, mutational analysis of *RAS* may, thus, provide an additional and readily available molecular tool for patients with MF to better predict their prognosis (already at early time points of disease) and to choose risk-adapted and/or personalized treatment strategies in the future. Motivated by our findings, further experimental studies are planned for investigating these molecular alterations on a more functional level in cell culture models of CTCL in order to pave the way for innovative treatment approaches in the future. 

## Figures and Tables

**Table 1:** Patient characteristics.

Pat ID	Sex	Year of First Diagnosis	Age at First Diagnosis (Years)	Stage of First Diagnosis	Previous History of Patches and Plaques Prior to First Diagnosis (Months)	Duration of Stage I (Patches and Plaques) Since First Diagnosis (Months)	Development of Skin Tumors	Time Since First Diagnosis until Development of Skin Tumors (Months)	Systemic Dissemination (Nodal/Visceral Site)	Time Since First Diagnosis until Systemic Dissemination (Months)	Follow-up since First Diagnosis (Months)	Final Stage at Date Last Seen	Final Status	Mutations in Epigenetic Modifiers	Mutations in JAK/STAT-Signaling	Mutations in RAS Genes
1	f	2014	75	IB	72	5	yes	5	no	na	36	IIB	alive with disease			
2	m	2017	65	IIB	48	0	yes	0	yes (LN)	36	36	IVA2	dead of disease	yes	yes	
3	m	2017	78	IB	48	5	yes	5	yes (lung)	4	48	IVB	alive with disease	yes	yes	
4	f	2006	46	IA	12	7	no	na	yes (LN)	84	108	IVB	dead of disease			
5	m	2015	51	IB	36	24	yes	24	no	na	72	IIB	alive with disease	yes		yes
6	m	2018	89	IB	24	18	yes	18	no	na	36	IIB	alive with disease	yes		
7	f	2005	39	IB	UNK	96	yes	96	yes	156	156	IVA2	lost to follow up	yes		yes
8	m	2012	74	IB	12	24	yes	24	no	na	36	IIB	dead of disease	yes		
9	f	2010	40	IB	UNK	96	yes	96	no	na	132	IIB	alive with disease	yes		yes
10	f	2015	78	IA	6	72	no	na	no	na	72	IB	alive with disease			
11	m	2015	56	IA	132	72	no	na	no	na	72	IB–IIB	alive with disease	yes	yes	
12	f	2003	60	IB	UNK	180	yes	180	no	na	204	IIB	alive with disease			
13	f	2012	35	IA	24	84	yes	96	yes (LN, liver)	84	99	IVB	alive with disease			
14	m	1995	41	IA	UNK	216	yes	216	no	na	312	IIB	alive with disease			
15	f	2015	73	IB	96	36	no	na	no	na	48	III	lost to follow up	yes		
16	m	2018	56	IIB	24	0	yes	0	yes (liver)	3	36	IVB	alive with disease			
17	f	2013	71	IIB	492	0	yes	0	no	na	48	IIB	dead of other cause			
18	m	2005	57	IIB	60	0	yes	0	no	na	60	IIB	lost to follow up		yes	
19	m	2011	62	IA	120	72	yes	72	no	na	120	IIB	alive without disease	yes		
20	f	2000	65	IA	UNK	84	yes	84	no	na	240	IIB	alive with disease			
21	m	2017	62	IB	180	48	no	na	no	na	48	IB	alive with disease			
22	m	2015	53	IB	60	72	no	na	no	na	72	IB	alive with disease			
23	m	2000	53	IA	UNK	252	no	na	no	na	252	IB	alive with disease			
24	m	2001	65	IA	UNK	240	no	na	no	na	240	IB	alive with disease			
25	m	2018	46	IA	360	36	no	na	no	na	36	IA	alive with disease			
26	f	1999	61	IA	UNK	348	no	na	no	na	348	IB	dead of other cause			
27	m	2019	59	IB	12	22	no	na	no	na	22	IB	alive with disease	yes		

Clinical data, disease course and mutational profile are depicted. NA: not applicable. LN: lymph node. UNK: unknown.

## Data Availability

Data will be provided upon request for reasonable academic studies from the corresponding author.

## Supplementary Materials

The following are available online at https://www.mdpi.com/article/10.3390/cancers13215512/s1, Table S1: Summary of applied treatment modalities of the 27 patients, Table S2: Statistical analysis of mutation occurrence in aggressive Mycosis fungoides with (group I) or without LCT (group II) in comparison to indolent Mycosis fungoides (group III), Table S3: Somatic variants identified with hybridization-based panel sequencing, Table S4: Hybridization-based panel sequencing read statistics.
